# Supplementary figures and images for: Cellular senescence-associated genes in rheumatoid arthritis: Identification and functional analysis
Source: PLoS One. 2025 Jan 16;20(1):e0317364. doi: 10.1371/journal.pone.0317364 (PMC11737674; doi:10.1371/journal.pone.0317364)

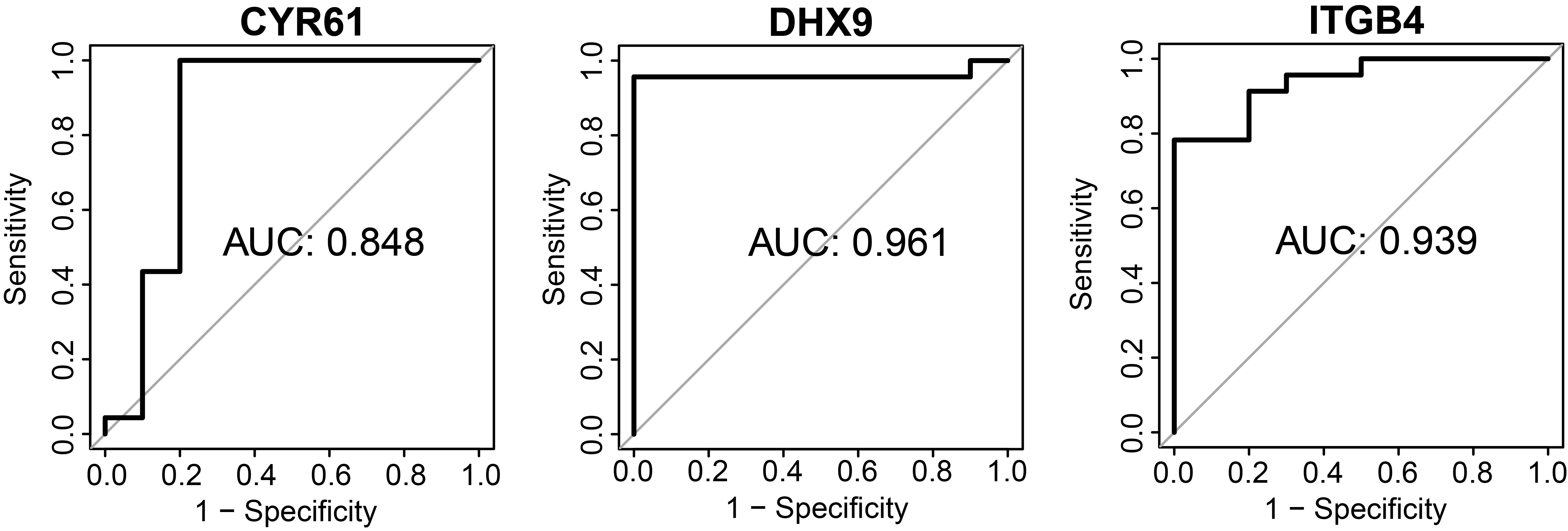

Supplement: S1 Fig — (TIF) [file pone.0317364.s001.tif]

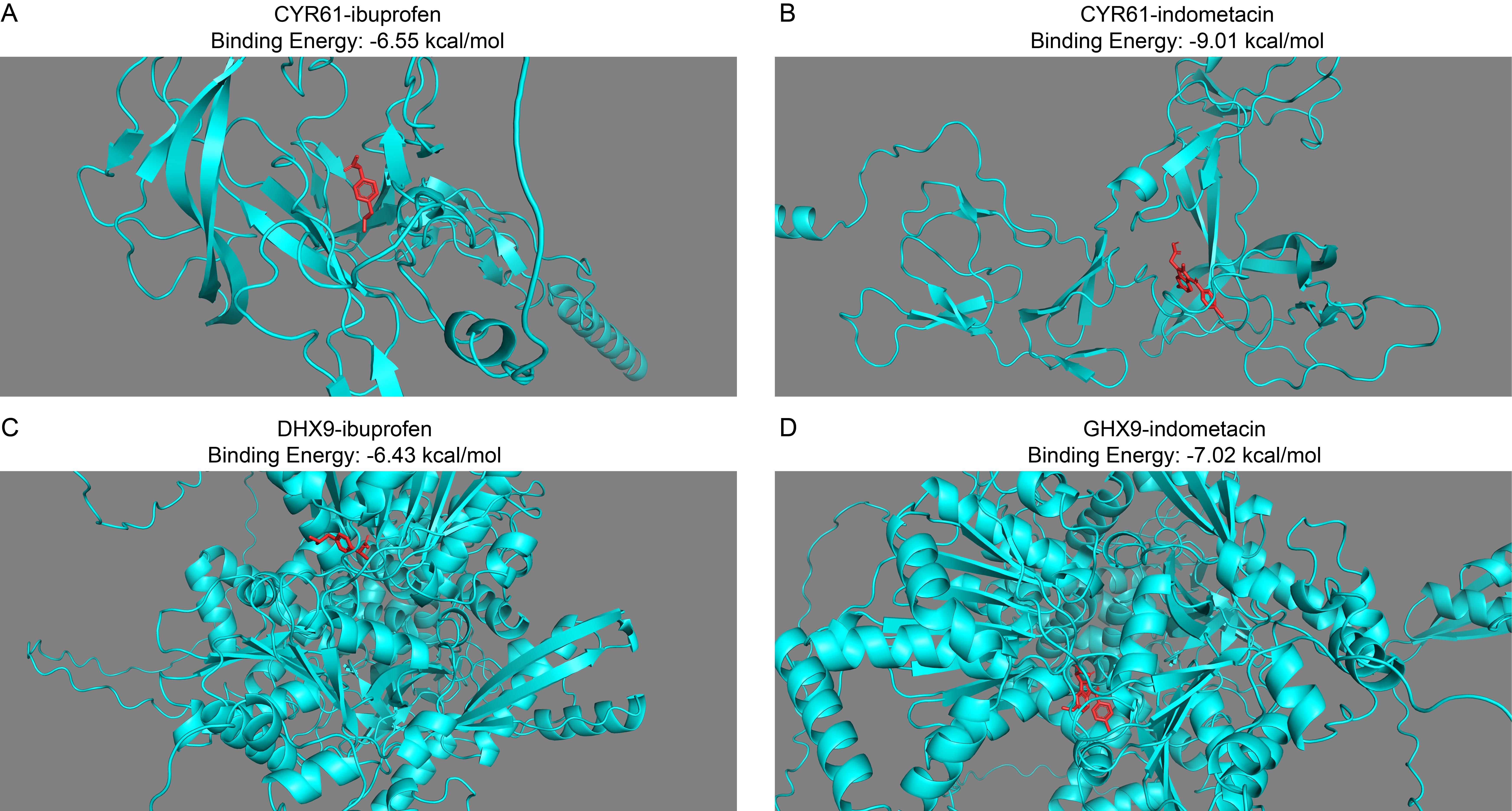

Supplement: S2 Fig — (TIF) [file pone.0317364.s002.tif]
