# Supplementary material for: Cellular senescence-associated genes in rheumatoid arthritis: Identification and functional analysis
Source: PLoS One. 2025 Jan 16;20(1):e0317364. doi: 10.1371/journal.pone.0317364 (PMC11737674; doi:10.1371/journal.pone.0317364)
Supplement: S1 Table — (DOCX) [file pone.0317364.s003.docx]

**S1 Table. The cellular senescence-related genes from CellAge database.**

| **Gene Symbol** | **ENTREZID** |  | **Gene Symbol** | **ENTREZID** |
| --- | --- | --- | --- | --- |
| ACLY | 47 |  | CDK18 | 5129 |
| AAK1 | 22848 |  | CDK2AP1 | 8099 |
| ABI3 | 51225 |  | CDK6 | 1021 |
| ADCK5 | 203054 |  | CDK4 | 1019 |
| AKR1B1 | 231 |  | CDKN1A | 1026 |
| AGT | 183 |  | CDKN1C | 1028 |
| AKT1 | 207 |  | CDKN1B | 1027 |
| ALOX15B | 247 |  | CDKN2A | 1029 |
| AR | 367 |  | CDKN2AIP | 55602 |
| ARPC1B | 10095 |  | CDKN2B | 1030 |
| ASF1A | 25842 |  | CENPA | 1058 |
| ASPH | 444 |  | CEBPB | 1051 |
| ATF7IP | 55729 |  | CHEK1 | 1111 |
| ATM | 472 |  | CKB | 1152 |
| AURKA | 6790 |  | CPEB1 | 64506 |
| AXL | 558 |  | CSNK1A1 | 1452 |
| BAG3 | 9531 |  | CTNNAL1 | 8727 |
| BHLHE40 | 8553 |  | CSNK2A1 | 1457 |
| BCL6 | 604 |  | CXCL1 | 2919 |
| BLK | 640 |  | DDB2 | 1643 |
| BLVRA | 644 |  | CYR61 | 3491 |
| BMI1 | 648 |  | DEK | 7913 |
| BRAF | 673 |  | DGCR8 | 54487 |
| BRD7 | 29117 |  | DHCR24 | 1718 |
| BRCA1 | 672 |  | DLX2 | 1746 |
| BTG3 | 10950 |  | DHX9 | 1660 |
| C11orf31 | 280636 |  | DPY30 | 84661 |
| CAV1 | 857 |  | DUSP3 | 1845 |
| CBX7 | 23492 |  | DUSP16 | 80824 |
| CBX8 | 57332 |  | E2F1 | 1869 |
| CCND1 | 595 |  | EHF | 26298 |
| CDK1 | 983 |  | ENDOG | 2021 |
| EPHA3 | 2042 |  | IGFBP3 | 3486 |
| ERRFI1 | 54206 |  | IGFBP6 | 3489 |
| ETS1 | 2113 |  | IGFBP5 | 3488 |
| ETS2 | 2114 |  | IL1A | 3552 |
| EWSR1 | 2130 |  | IL8 | 3576 |
| FASTK | 10922 |  | ING1 | 3621 |
| EZH2 | 2146 |  | ING2 | 3622 |
| FBXO31 | 79791 |  | IRF3 | 3661 |
| FOXM1 | 2305 |  | IRF5 | 3663 |
| FOS | 2353 |  | IRF7 | 3665 |
| FOXO3 | 2309 |  | ITPK1 | 3705 |
| FXR1 | 8087 |  | ITGB4 | 3691 |
| G6PD | 2539 |  | ITPKB | 3707 |
| GAPDH | 2597 |  | ITSN2 | 50618 |
| GKN1 | 56287 |  | KCNJ12 | 3768 |
| GATA4 | 2626 |  | KDM4A | 9682 |
| GNG11 | 2791 |  | KDM5B | 10765 |
| GLB1 | 2720 |  | KIAA1524 | 57650 |
| GRK6 | 2870 |  | KL | 9365 |
| HDAC4 | 9759 |  | KSR2 | 283455 |
| HDAC1 | 3065 |  | LATS1 | 9113 |
| HEPACAM | 220296 |  | LEO1 | 123169 |
| HJURP | 55355 |  | LGALS3 | 3958 |
| HIVEP1 | 3096 |  | LIMA1 | 51474 |
| HK3 | 3101 |  | LIMK1 | 3984 |
| HMGB1 | 3146 |  | MAGEA2 | 4101 |
| HRAS | 3265 |  | MAGOH | 4116 |
| HSPA5 | 3309 |  | MAD2L1 | 4085 |
| HSPB2 | 3316 |  | MAGOHB | 55110 |
| ID1 | 3397 |  | MAP2K1 | 5604 |
| ID4 | 3400 |  | MAP2K3 | 5606 |
| IGFBP1 | 3484 |  | MAP2K2 | 5605 |
| IFNG | 3458 |  | MAP2K6 | 5608 |
| MAP3K6 | 9064 |  | NTN4 | 59277 |
| MAP2K7 | 5609 |  | NUAK1 | 9891 |
| MAP4K1 | 11184 |  | OTX2 | 5015 |
| MAP3K7 | 6885 |  | P3H1 | 64175 |
| MAPK12 | 6300 |  | PATZ1 | 23598 |
| MAPKAPK5 | 8550 |  | PAK4 | 10298 |
| 5-Mar | 54708 |  | PBRM1 | 55193 |
| MAPK14 | 1432 |  | PCGF2 | 7703 |
| MAST1 | 22983 |  | PDCD10 | 11235 |
| MATK | 4145 |  | PDIK1L | 149420 |
| MCL1 | 4170 |  | PDZD2 | 23037 |
| MDH1 | 4190 |  | PDPK1 | 5170 |
| MCRS1 | 10445 |  | PEBP1 | 5037 |
| MECP2 | 4204 |  | PEX19 | 5824 |
| MOB3A | 126308 |  | PIAS4 | 51588 |
| MMP9 | 4318 |  | PIK3R5 | 23533 |
| MORC3 | 23515 |  | PIK3C2A | 5286 |
| MORF4 | 10934 |  | PIM1 | 5292 |
| MXD4 | 10608 |  | PLA2R1 | 22925 |
| MVK | 4598 |  | PKM | 5315 |
| MYC | 4609 |  | PML | 5371 |
| MYLK | 4638 |  | PNPT1 | 87178 |
| NADK | 65220 |  | PMVK | 10654 |
| NANOG | 79923 |  | POT1 | 25913 |
| NDRG1 | 10397 |  | POU5F1 | 5460 |
| NEK1 | 4750 |  | PPM1B | 5495 |
| NEK4 | 6787 |  | PPM1D | 8493 |
| NEK6 | 10783 |  | PRMT6 | 55170 |
| NFE2L2 | 4780 |  | PRKCH | 5583 |
| NINJ1 | 4814 |  | PRKCD | 5580 |
| NOTCH3 | 4854 |  | PROX1 | 5629 |
| NOX4 | 50507 |  | PRPF19 | 27339 |
| NR2E1 | 7101 |  | PSMB5 | 5693 |
| PTRF | 284119 |  | SORBS2 | 8470 |
| PTTG1 | 9232 |  | SOX2 | 6657 |
| PSMD14 | 10213 |  | SPIN1 | 10927 |
| RAD21 | 5885 |  | SOX5 | 6660 |
| RAF1 | 5894 |  | SP1 | 6667 |
| RB1 | 5925 |  | SPOP | 8405 |
| RBP2 | 5948 |  | SRC | 6714 |
| RBX1 | 9978 |  | SREBF1 | 6720 |
| RNASEL | 6041 |  | SRSF1 | 6426 |
| RPS6KA6 | 27330 |  | STAT5B | 6777 |
| RSL1D1 | 26156 |  | STK32C | 282974 |
| RUNX1 | 861 |  | STK40 | 83931 |
| RUVBL2 | 10856 |  | SUPT5H | 6829 |
| SENP1 | 29843 |  | SYK | 6850 |
| SENP2 | 59343 |  | TACC3 | 10460 |
| SENP7 | 57337 |  | TERC | 7012 |
| SERPINE1 | 5054 |  | TBX2 | 6909 |
| SFN | 2810 |  | TERF2 | 7014 |
| SIK1 | 150094 |  | TERT | 7015 |
| SGK1 | 6446 |  | TFAP4 | 7023 |
| SIN3B | 23309 |  | TFDP1 | 7027 |
| SIRT1 | 23411 |  | TGFB1I1 | 7041 |
| SIRT6 | 51548 |  | TLR3 | 7098 |
| SIX1 | 6495 |  | TMSB4X | 7114 |
| SLC13A3 | 64849 |  | TNFSF13 | 8741 |
| SLC16A7 | 9194 |  | TNFSF15 | 9966 |
| SMARCA4 | 6597 |  | TOP1 | 7150 |
| SMG1 | 23049 |  | TP63 | 8626 |
| SMARCB1 | 6598 |  | TPR | 7175 |
| SMURF2 | 64750 |  | TP53 | 7157 |
| SNAI1 | 6615 |  | TRIM28 | 10155 |
| SOCS1 | 8651 |  | TRPM8 | 79054 |
| SOD1 | 6647 |  | TXN | 7295 |
| TXNIP | 10628 |  | WRN | 7486 |
| UBTD1 | 80019 |  | WT1 | 7490 |
| TYK2 | 7297 |  | XAF1 | 54739 |
| VENTX | 27287 |  | WWP1 | 11059 |
| USP1 | 7398 |  | YAP1 | 10413 |
| VEGFA | 7422 |  | YPEL3 | 83719 |
| WNT16 | 51384 |  | ZFP36 | 7538 |
| WNT2 | 7472 |  | ZMAT3 | 64393 |
