# Supplementary material for: Cellular senescence-associated genes in rheumatoid arthritis: Identification and functional analysis
Source: PLoS One. 2025 Jan 16;20(1):e0317364. doi: 10.1371/journal.pone.0317364 (PMC11737674; doi:10.1371/journal.pone.0317364)
Supplement: S2 Table — (DOCX) [file pone.0317364.s004.docx]

**S2 Table. The cellular senescence related DEGs between RA and normal samples.**

| **Gene Symbol** | **logFC** | **P.Value** | **UP/Down** |
| --- | --- | --- | --- |
| DHX9 | 2.19701593 | 8.50E-07 | Up |
| SOX5 | 1.611499638 | 0.014050375 | Up |
| CYR61 | 1.419352065 | 9.54E-05 | Up |
| SP1 | 1.263011655 | 0.001141202 | Up |
| SNAI1 | 1.102811457 | 0.000351434 | Up |
| ITGB4 | 1.090114172 | 1.83E-05 | Up |
| MAP2K7 | 1.068325939 | 0.003221883 | Up |
| SLC16A7 | 1.037477885 | 0.000164989 | Up |
| SOX2 | 0.935646024 | 0.001583482 | Up |
| CBX8 | 0.86597859 | 0.010305009 | Up |
| SRC | 0.730835715 | 0.036619745 | Up |
| SPIN1 | 0.70202799 | 0.0025421 | Up |
| MVK | 0.677844256 | 0.014698101 | Up |
| ASPH | 0.661162921 | 0.000534449 | Up |
| SGK1 | 0.639690108 | 0.012613697 | Up |
| AR | 0.595826441 | 0.008057821 | Up |
| SIN3B | 0.547666218 | 7.46E-05 | Up |
| DUSP3 | 0.51511554 | 0.010993302 | Up |
| SMARCA4 | 0.504097203 | 0.001965883 | Up |
| CDK1 | -0.522282517 | 0.016825973 | Down |
| SYK | -0.539002108 | 0.027552231 | Down |
| NEK4 | -0.587699694 | 0.001714848 | Down |
| TNFSF13 | -0.643526318 | 0.017808327 | Down |
| ASF1A | -0.644173167 | 0.000177922 | Down |
| DDB2 | -0.659388947 | 0.008404035 | Down |
| BLVRA | -0.719365205 | 0.000105404 | Down |
| AURKA | -0.74494127 | 0.025146166 | Down |
| MATK | -0.821336687 | 0.044719437 | Down |
| TLR3 | -0.995872471 | 0.000443145 | Down |
