# Supplementary material for: Cellular senescence-associated genes in rheumatoid arthritis: Identification and functional analysis
Source: PLoS One. 2025 Jan 16;20(1):e0317364. doi: 10.1371/journal.pone.0317364 (PMC11737674; doi:10.1371/journal.pone.0317364)
Supplement: S3 Table — (DOCX) [file pone.0317364.s005.docx]

**S3 Table. The GO terms that the cellular senescence related DEGs involved in.**

| **ID** | **Description** | **P value** | **GO** |
| --- | --- | --- | --- |
| GO:0051054 | positive regulation of DNA metabolic process | 4.35E-06 | BP |
| GO:0051090 | regulation of DNA-binding transcription factor activity | 4.46E-05 | BP |
| GO:0019083 | viral transcription | 5.78E-05 | BP |
| GO:0038093 | Fc receptor signaling pathway | 7.25E-05 | BP |
| GO:0043410 | positive regulation of MAPK cascade | 7.30E-05 | BP |
| GO:0018105 | peptidyl-serine phosphorylation | 8.66E-05 | BP |
| GO:0032481 | positive regulation of type I interferon production | 0.000108902 | BP |
| GO:0018209 | peptidyl-serine modification | 0.00011432 | BP |
| GO:0070371 | ERK1 and ERK2 cascade | 0.000127819 | BP |
| GO:0033143 | regulation of intracellular steroid hormone receptor signaling pathway | 0.000162103 | BP |
| GO:2000241 | regulation of reproductive process | 0.00022846 | BP |
| GO:0071383 | cellular response to steroid hormone stimulus | 0.000241427 | BP |
| GO:0043923 | positive regulation by host of viral transcription | 0.000283824 | BP |
| GO:2000243 | positive regulation of reproductive process | 0.000283847 | BP |
| GO:0050727 | regulation of inflammatory response | 0.000318945 | BP |
| GO:0045780 | positive regulation of bone resorption | 0.000319009 | BP |
| GO:0071360 | cellular response to exogenous dsRNA | 0.000319009 | BP |
| GO:0007044 | cell-substrate junction assembly | 0.000367741 | BP |
| GO:2000116 | regulation of cysteine-type endopeptidase activity | 0.000377041 | BP |
| GO:0032755 | positive regulation of interleukin-6 production | 0.000415069 | BP |
| GO:0019080 | viral gene expression | 0.000427483 | BP |
| GO:0150115 | cell-substrate junction organization | 0.000440133 | BP |
| GO:0001649 | osteoblast differentiation | 0.000448231 | BP |
| GO:0002223 | stimulatory C-type lectin receptor signaling pathway | 0.000479879 | BP |
| GO:1990840 | response to lectin | 0.000479879 | BP |
| GO:1990858 | cellular response to lectin | 0.000479879 | BP |
| GO:0032479 | regulation of type I interferon production | 0.000493136 | BP |
| GO:0032606 | type I interferon production | 0.000493136 | BP |
| GO:0032760 | positive regulation of tumor necrosis factor production | 0.000493136 | BP |
| GO:1901655 | cellular response to ketone | 0.000493136 | BP |
| GO:0000792 | heterochromatin | 0.00538289 | CC |
| GO:0005876 | spindle microtubule | 0.006653591 | CC |
| GO:0031234 | extrinsic component of cytoplasmic side of plasma membrane | 0.008538486 | CC |
| GO:0000805 | X chromosome | 0.014006425 | CC |
| GO:0099091 | postsynaptic specialization, intracellular component | 0.014006425 | CC |
| GO:0140092 | bBAF complex | 0.014006425 | CC |
| GO:0032009 | early phagosome | 0.016784919 | CC |
| GO:0098687 | chromosomal region | 0.016861849 | CC |
| GO:0001650 | fibrillar center | 0.017658998 | CC |
| GO:0031464 | Cul4A-RING E3 ubiquitin ligase complex | 0.018171333 | CC |
| GO:0044292 | dendrite terminus | 0.018171333 | CC |
| GO:0001741 | XY body | 0.019555861 | CC |
| GO:0016586 | RSC-type complex | 0.019555861 | CC |
| GO:0031616 | spindle pole centrosome | 0.019555861 | CC |
| GO:0045120 | pronucleus | 0.019555861 | CC |
| GO:0071564 | npBAF complex | 0.019555861 | CC |
| GO:0072687 | meiotic spindle | 0.019555861 | CC |
| GO:0140288 | GBAF complex | 0.019555861 | CC |
| GO:0016607 | nuclear speck | 0.020640875 | CC |
| GO:0035102 | PRC1 complex | 0.020938507 | CC |
| GO:0005819 | spindle | 0.021554042 | CC |
| GO:0001674 | female germ cell nucleus | 0.022319273 | CC |
| GO:0071565 | nBAF complex | 0.022319273 | CC |
| GO:0019897 | extrinsic component of plasma membrane | 0.024302092 | CC |
| GO:0009898 | cytoplasmic side of plasma membrane | 0.024565148 | CC |
| GO:0072686 | mitotic spindle | 0.027260021 | CC |
| GO:0036020 | endolysosome membrane | 0.027823582 | CC |
| GO:0016580 | Sin3 complex | 0.030564519 | CC |
| GO:0070822 | Sin3-type complex | 0.030564519 | CC |
| GO:0030496 | midbody | 0.032989653 | CC |
| GO:0004674 | protein serine/threonine kinase activity | 3.79E-05 | MF |
| GO:0004712 | protein serine/threonine/tyrosine kinase activity | 4.27E-05 | MF |
| GO:0004715 | non-membrane spanning protein tyrosine kinase activity | 4.27E-05 | MF |
| GO:0004713 | protein tyrosine kinase activity | 5.36E-05 | MF |
| GO:0061629 | RNA polymerase II-specific DNA-binding transcription factor binding | 0.000164577 | MF |
| GO:0106310 | protein serine kinase activity | 0.000195031 | MF |
| GO:0035173 | histone kinase activity | 0.000415722 | MF |
| GO:0043274 | phospholipase binding | 0.000552009 | MF |
| GO:0042393 | histone binding | 0.000589401 | MF |
| GO:0001221 | transcription coregulator binding | 0.000591743 | MF |
| GO:0140297 | DNA-binding transcription factor binding | 0.000676212 | MF |
| GO:0005178 | integrin binding | 0.001667893 | MF |
| GO:0042169 | SH2 domain binding | 0.001759141 | MF |
| GO:0140030 | modification-dependent protein binding | 0.002203543 | MF |
| GO:0008022 | protein C-terminus binding | 0.002546783 | MF |
| GO:0061980 | regulatory RNA binding | 0.00270972 | MF |
| GO:0140677 | molecular function activator activity | 0.003609179 | MF |
| GO:0001098 | basal transcription machinery binding | 0.003852973 | MF |
| GO:0001099 | basal RNA polymerase II transcription machinery binding | 0.003852973 | MF |
| GO:0097110 | scaffold protein binding | 0.004495093 | MF |
| GO:0003725 | double-stranded RNA binding | 0.005916866 | MF |
| GO:0035064 | methylated histone binding | 0.006535956 | MF |
| GO:0140034 | methylation-dependent protein binding | 0.006535956 | MF |
| GO:0051117 | ATPase binding | 0.007349535 | MF |
| GO:0003727 | single-stranded RNA binding | 0.008031758 | MF |
| GO:0051219 | phosphoprotein binding | 0.008561498 | MF |
| GO:0031490 | chromatin DNA binding | 0.01378002 | MF |
| GO:0008094 | ATP-dependent activity, acting on DNA | 0.014452203 | MF |
| GO:0001163 | RNA polymerase I transcription regulatory region sequence-specific DNA binding | 0.015091207 | MF |
| GO:0001164 | RNA polymerase I core promoter sequence-specific DNA binding | 0.015091207 | MF |
