# Supplementary material for: Cellular senescence-associated genes in rheumatoid arthritis: Identification and functional analysis
Source: PLoS One. 2025 Jan 16;20(1):e0317364. doi: 10.1371/journal.pone.0317364 (PMC11737674; doi:10.1371/journal.pone.0317364)
Supplement: S4 Table — (DOCX) [file pone.0317364.s006.docx]

**S4 Table. The KEGG pathways that the cellular senescence related DEGs involved in.**

| **ID** | **Description** | **pvalue** | **p.adjust** |
| --- | --- | --- | --- |
| hsa05161 | Hepatitis B | 0.000510849 | 0.000510849 |
| hsa05167 | Kaposi sarcoma-associated herpesvirus infection | 0.00100639 | 0.00100639 |
| hsa04114 | Oocyte meiosis | 0.003497683 | 0.003497683 |
| hsa04664 | Fc epsilon RI signaling pathway | 0.011205877 | 0.011205877 |
| hsa05169 | Epstein-Barr virus infection | 0.011606555 | 0.011606555 |
| hsa05203 | Viral carcinogenesis | 0.01192023 | 0.01192023 |
| hsa04137 | Mitophagy - animal | 0.01250202 | 0.01250202 |
| hsa04115 | p53 signaling pathway | 0.01283591 | 0.01283591 |
| hsa04012 | ErbB signaling pathway | 0.017141853 | 0.017141853 |
| hsa04540 | Gap junction | 0.018302479 | 0.018302479 |
| hsa04520 | Adherens junction | 0.020309259 | 0.020309259 |
| hsa04912 | GnRH signaling pathway | 0.020309259 | 0.020309259 |
| hsa01522 | Endocrine resistance | 0.02240464 | 0.02240464 |
| hsa04914 | Progesterone-mediated oocyte maturation | 0.024143283 | 0.024143283 |
| hsa04620 | Toll-like receptor signaling pathway | 0.025033007 | 0.025033007 |
| hsa04625 | C-type lectin receptor signaling pathway | 0.025033007 | 0.025033007 |
| hsa04722 | Neurotrophin signaling pathway | 0.032124556 | 0.032124556 |
| hsa04611 | Platelet activation | 0.034646554 | 0.034646554 |
| hsa04380 | Osteoclast differentiation | 0.036718774 | 0.036718774 |
| hsa04926 | Relaxin signaling pathway | 0.037244284 | 0.037244284 |
| hsa05135 | Yersinia infection | 0.0415533 | 0.0415533 |
| hsa04915 | Estrogen signaling pathway | 0.042104813 | 0.042104813 |
| hsa05418 | Fluid shear stress and atherosclerosis | 0.04265914 | 0.04265914 |
| hsa05224 | Breast cancer | 0.047193232 | 0.047193232 |
| hsa04151 | PI3K-Akt signaling pathway | 0.049933407 | 0.049933407 |
