# Supplementary material for: Cellular senescence-associated genes in rheumatoid arthritis: Identification and functional analysis
Source: PLoS One. 2025 Jan 16;20(1):e0317364. doi: 10.1371/journal.pone.0317364 (PMC11737674; doi:10.1371/journal.pone.0317364)
Supplement: Attachment — Submitted filename: review_PONE-D-24-02627_R1.docx [file pone.0317364.s007.docx]

**Dear Editor and Authors,**

**Overall Assessment**

I highly value the quality and findings presented in the manuscript and its intentions, objectives, and methodology. The strategy followed is appropriate, and the manuscript is worthy of publication in PLOS One. However, several aspects require significant major revision to strengthen the study further.

**Methodology of Random Forest**

While the use of Random Forest methodology has been relatively successful in developing a decision network, it has certain limitations that should be explored within the context of the study. Even though the ROC results presented are excellent, it is essential to address the possibility that the set of 29 genes identified may be incomplete and some relevant genes might have been overlooked. In this regard, comparing the predictive capabilities with an XGBoost model would be beneficial. XGBoost allows for monitoring and learning according to a loss function, potentially offering better performance in identifying additional relevant genes. Additionally, XGBoost could enhance the detection of the 29 previously selected genes by providing:

*Enhanced Sensitivity and Specificity:*

By optimizing function loss more effectively, XGBoost could potentially improve the sensitivity and specificity of gene detection, ensuring that more relevant genes are identified without increasing false positives.

*Better Handling of Feature Interactions:*

XGBoost can more effectively capture complex interactions between genes than Random Forest. This could lead to a more accurate identification of the key genes involved in the disease process.

*Increased Robustness:*

Through its regularization parameters, XGBoost reduces the risk of overfitting, ensuring that the identified genes are relevant and not artifacts of the training data.

**Docking Studies**

The proposed docking studies are a good starting point, but I identify several needs that are important to address:

*Reference to AutoDock Procedure:*

Appropriately referencing the AutoDock procedure is essential. Extensive, updated, and relevant literature on this topic exists, and a proper reference reflecting the use of the chosen suite is necessary.

*Protein Structures:*

The protein structures encoding the selected genes were obtained from PDB and possibly other sources. Given the advantages and excellent results provided by AlphaFold, I recommend that the authors compare the secondary structure of the proteins encoded by these genes with those found in PDB using AlphaFold predictions. This could offer additional validation and improve structural accuracy.

*Topological Parameters of Ligands*

Although the selected ligands are biochemically appropriate, it would be interesting to detail how the topological parameters of these ligands were obtained. If these parameters were resolved using DFT (Density Functional Theory), it is essential to specify which bases or functionals were used. Alternatively, the specific force field used and the rationale behind its selection should be explained if force fields were employed.

*Molecular Dynamics Simulations:*

While the AutoDock results are appropriate and relatively solid, I recommend that the authors conduct additional molecular dynamics simulations. Using an effective platform such as GROMACS for these simulations could provide valuable insights into the stability of the formed complex. Molecular dynamics simulations would allow for observing how the docking complex behaves in a simulated environment and verifying its stability over time.

**Conclusion**

The manuscript presents significant findings and utilizes robust methodologies, but it would benefit from the proposed improvements and major revisions. Implementing these recommendations could strengthen the study's results and conclusions, providing additional validation and a deeper understanding of the underlying biological mechanisms.
