# Supplementary material for: Cellular senescence-associated genes in rheumatoid arthritis: Identification and functional analysis
Source: PLoS One. 2025 Jan 16;20(1):e0317364. doi: 10.1371/journal.pone.0317364 (PMC11737674; doi:10.1371/journal.pone.0317364)
Supplement: Attachment — Submitted filename: response-to-reviewers.docx [file pone.0317364.s008.docx]

Dear Prof. Gurudeeban Selvaraj and reviewers:

Thank you very much for giving us another opportunity to revise and resubmit our manuscript, we appreciate editor and reviewers very much for their positive and constructive comments and suggestions on our manuscript entitled “Cellular Senescence-Associated Genes in Rheumatoid Arthritis: Identification and Functional Analysis” (PONE-D-24-02627). Those comments are all valuable and helpful for improving our paper. We have studied reviewer’s comment carefully and have made revision which marked in red in the paper. And we have tried our best to revise our manuscript according to the comments. The main corrections in the paper and the response to the reviewers’ comments are as follows:

**Comments to the Author**

*4. Have the authors* *made all data underlying the findings in their manuscript fully available?*

*The PLOS Data policy requires authors to make all data underlying the findings described in their manuscript fully available without restriction, with rare exception (please refer to the Data Availability Statement in the manuscript PDF file). The data should be provided as part of the manuscript or its supporting information, or deposited to a public repository. For example, in addition to summary statistics, the data points behind means, medians and variance measures should be available. If there are restrictions on publicly sharing data—e.g. participant privacy or use of data from a third party—those must be specified.*

*Reviewer #1: Yes*

*Reviewer #2: No*

**REPLY:** Thank you very much for your comments. In our manuscript, five rheumatoid arthritis datasets were used, including GSE55457, GSE12021, GSE55235, GSE77298, and GSE178557. And all the datasets were downloaded from GEO database. The comprehensive details of these five cohorts are delineated in Table 1 of our study. In parallel, the specifics of these genes are cataloged in Supplementary Table S1 of our documentation. That’s to say, we have made all data underlying the findings in our manuscript fully available. If the reviewer thinks that we still need to provide more detailed information, we will be glad to do so.

*6. Review Comments to the Author*

*Please use the space provided to explain your answers to the questions above. You may also include additional comments for the author, including concerns about dual publication, research ethics, or publication ethics. (Please upload your review as an attachment if it exceeds 20,000 characters)*

*Reviewer #1: (No Response)*

*Reviewer #2: Dear Editor and Authors,*

*Overall Assessment*

*I highly value the quality and findings presented in the manuscript and its intentions, objectives, and methodology. The strategy followed is appropriate, and the manuscript is worthy of publication in PLOS One. However, several aspects require significant major revision to strengthen the study further.*

**REPLY:** Thank you very much for your further modification comments. We have studied your comment carefully and have made revision to meet PLOS ONE’s publication criteria.

*Methodology of Random Forest*

*While the use of Random Forest methodology has been relatively successful in developing adecision network, it has certain limitations that should be explored within the context of the study. Even though the ROC results presented are excellent, it is essential to address the possibility that the set of 29 genes identified may be incomplete and some relevant genes might have been overlooked. In this regard, comparing the predictive capabilities with an XGBoost model would be beneficial. XGBoost allows for monitoring and learning according to a loss function, potentially offering better performance in identifying additional relevant genes. Additionally, XGBoost could enhance the detection of the 29 previously selected genes by providing:*

*Enhanced Sensitivity and Specificity:*

*By optimizing function loss more effectively, XGBoost could potentially improve the sensitivity and specificity of gene detection, ensuring that more relevant genes are identified without increasing false positives.*

*Better Handling of Feature Interactions:*

*XGBoost can more effectively capture complex interactions between genes than Random Forest. This could lead to a more accurate identification of the key genes involved in the disease process.*

*Increased Robustness:*

*Through its regularization parameters, XGBoost reduces the risk of overfitting, ensuring that the identified genes are relevant and not artifacts of the training data.*

**REPLY:** Thank you very much for your further modification comments. In our manuscript, differential expressed analysis was first carried out between RA and normal samples. In total, there were 2050 DEGs identified using the filtering criteria of P-value less than 0.05 and the absolute value of log2 FC greater than 0.5 (Figure 2A). Next, we investigated the differential expression of cellular senescence-related genes and found that 29 of the 279 cellular senescence-related genes were significantly differentially expressed (Figure 2B). Subsequently, the random forest algorithm and LASSO regression analysis were respectively performed. The results showed that there were 3 cellular senescence-related DEGs identified by random forest algorithm and LASSO regression analysis respectively (Figure 2C-D). Then, 3 out of the 29 cellular senescence-related DEGs were ultimately remained as hub genes, including DHX9, CYR61 and ITGB (Figure 2C-D). According to your suggestions, we also performed XGBoost based on the expression levels of 29 cellular senescence-related genes after differential expression analysis. And our analysis found that there are 12 genes remained through XGBoost algorithm, but through LASSO regression analysis, there are still 3 cellular senescence-related DEGs retained, including DHX9, CYR61 and ITGB. This result shows that although XGBoost algorithm can retain more relevant genes, after LASSO algorithm, only three genes are still retained for model construction, which is consistent with the result of random forest. On the other hand, the purpose of our study is to detect fewer signature genes to distinguish between RA and normal samples, thus reducing the detection cost. Therefore, in this study, we are inclined to use random forest to further screen cellular senescence related genes that are more relevant to RA.

*Docking Studies*

*The proposed docking studies are a good starting point, but I identify several needs that are important to address:*

*Reference to AutoDock Procedure:*

*Appropriately referencing the AutoDock procedure is essential. Extensive, updated, and relevant literature on this topic exists, and a proper reference reflecting the use of the chosen suite is necessary.*

**REPLY:** Thank you very much for your further modification comments. According to your suggestions, we have appropriately reference the AutoDock procedure in our manuscript, and we have revised the manuscript as follows:

Page 8-9, section “**Association between signature genes and drug sensitivity**” in **Materials and Methods**

“To further explore whether the signature genes we identified could be potential targets for these retrieved rheumatoid arthritis drugs, AutoDock was performed. Detailedly, the crystal structures of signature genes were downloaded from RCSB Protein Data Bank (PDB, https://www.rcsb.org/) and the 3D structure of small molecule compound was downloaded from the PubChem Compound database. The downloaded complex were embellished by PyMol2.3.0 to remove original water molecules and phosphates. Moreover, the AutoDock Tools 4.2.6 [PMID: 19399780] (https://autodock.scripps.edu/) was used to prepare receptors, including adding Gasteiger charges, merging non-polar hydrogen bonds and setting docking parameters. A box of 60*60*60-point grid (0.375-Å spacing between the grid points) and the affinity maps were generated and processed using AutoGrid 4.2 by default setting. For each docking case, 200 Lamarckian genetic algorithm runs were processed by default setting using AutoDock 4.2.6. To further confirm the binding of small molecule compound to the selected genes, we have also obtained the structures of proteins encoded by the selected genes from AlphaFold and then re-performed AutoDock. The top-scored hit was chosen and visualized for further analysis and the PyMOL was used to preparse all the molecular graphics.”

*Protein Structures:*

*The protein structures encoding the selected genes were obtained from PDB and possibly other sources. Given the advantages and excellent results provided by* *AlphaFold, I recommend that the authors compare the secondary structure of the proteins encoded by these genes with those found in PDB using AlphaFold predictions. This could* *offer additional validation and improve structural accuracy.*

**REPLY:** Thank you for your constructive comments. According to your suggestions, to further confirm the binding of small molecule compound to the selected genes, we have also obtained the structures of proteins encoded by the selected genes from AlphaFold and then re-performed AutoDock. As shown by the docking results, ibuprofen and indometacin both possessed high binding affinity with an average of -7.25 kcal/mol. Specifically, the binding energy of gene CYR61 and ibuprofen is -6.55 kcal/mol (Supplementary Figure S2A), while the binding energy of gene CYR61 and indometacin is -9.01 kcal/mol (Supplementary Figure S2B). Moreover, the binding energy of gene DHX9 and ibuprofen is -6.43 kcal/mol (Supplementary Figure S2C), while the binding energy of gene DHX9 and indometacin is -7.02 kcal/mol (Supplementary Figure S2D). There findings offered additional validation that the signature genes (CYR61 and DHX9) could be used as potential drug targets for RA treatment.

We have revised the manuscript as follows:

Page 8-9, section “**Association between signature genes and drug sensitivity**” in **Materials and Methods**

“To further explore whether the signature genes we identified could be potential targets for these retrieved rheumatoid arthritis drugs, AutoDock was performed. Detailedly, the crystal structures of signature genes were downloaded from RCSB Protein Data Bank (PDB, https://www.rcsb.org/) and the 3D structure of small molecule compound was downloaded from the PubChem Compound database. The downloaded complex were embellished by PyMol2.3.0 to remove original water molecules and phosphates. Moreover, the AutoDock Tools 4.2.6 [PMID: 19399780] (https://autodock.scripps.edu/) was used to prepare receptors, including adding Gasteiger charges, merging non-polar hydrogen bonds and setting docking parameters. A box of 60*60*60-point grid (0.375-Å spacing between the grid points) and the affinity maps were generated and processed using AutoGrid 4.2 by default setting. For each docking case, 200 Lamarckian genetic algorithm runs were processed by default setting using AutoDock 4.2.6. To further confirm the binding of small molecule compound to the selected genes, we have also obtained the structures of proteins encoded by the selected genes from AlphaFold and then re-performed AutoDock. The top-scored hit was chosen and visualized for further analysis and the PyMOL was used to preparse all the molecular graphics.”

Page 14-15, section “**Association between signature genes and drug sensitivity**” in **Results**

“To further confirm the binding of small molecule compound to the selected genes, we have also obtained the structures of proteins encoded by the selected genes from AlphaFold and then re-performed AutoDock. As shown by the docking results, ibuprofen and indometacin both possessed high binding affinity with an average of -7.25 kcal/mol. Specifically, the binding energy of gene CYR61 and ibuprofen is -6.55 kcal/mol (Supplementary Figure S2A), while the binding energy of gene CYR61 and indometacin is -9.01 kcal/mol (Supplementary Figure S2B). Moreover, the binding energy of gene DHX9 and ibuprofen is -6.43 kcal/mol (Supplementary Figure S2C), while the binding energy of gene DHX9 and indometacin is -7.02 kcal/mol (Supplementary Figure S2D). There findings suggested that the signature genes (CYR61 and DHX9) could be used as potential drug targets for RA treatment.”

*Topological Parameters of Ligands*

*Although the selected ligands are biochemically appropriate, it would be interesting to detail how the topological parameters of these ligands were obtained. If these parameters were resolved using DFT (Density Functional Theory), it is essential to specify which bases or functionals were used. Alternatively, the specific force field used and the rationale behind its selection should be explained if force fields were employed.*

**REPLY:** We appreciate the reviewer’s insightful suggestion to elaborate on the determination of the topological parameters of the selected ligands. In our study, these parameters were derived using the AutoDock suite, specifically employing AutoDockTools, which are optimized for ligand preparation in docking simulations. Detailed Ligand Preparation and Parameterization as follows:

- **Hydrogen Addition:** All ligand structures were first processed to ensure that hydrogen atoms were correctly added. This step is crucial for accurate representation of the ligand’s geometry and for subsequent charge calculations.
- **Charge Assignment:** Gasteiger charges were computed for all ligand atoms. The Gasteiger method, implemented in AutoDockTools, is a well-established approach for assigning partial atomic charges, which are critical for the electrostatic component of the docking scoring function.
- **Atom Typing:** Atom types were assigned according to the AutoDock parameterization scheme. This ensures that all atoms in the ligands are compatible with the scoring functions used by AutoDock.

And the selection of AutoDockTools for ligand parameterization was based on several key considerations:

- **Integration and Compatibility:** AutoDockTools is specifically designed to integrate seamlessly with the AutoDock docking engine, ensuring that all topological parameters are fully compatible with the scoring functions and algorithms used in the docking simulations.
- **Optimization for Docking:** The parameterization methods in AutoDockTools, such as the Gasteiger charge assignment and specific atom typing, are optimized for the accurate prediction of ligand-receptor interactions within the AutoDock framework.
- **Efficiency:** AutoDockTools provides an automated and efficient workflow for ligand preparation, which is particularly advantageous for handling multiple ligands in high-throughput docking studies.

**Justification Against DFT and Force Fields**

While Density Functional Theory (DFT) and specific force fields offer high precision in topological parameterization, the practical considerations of our study necessitated the use of AutoDockTools:

- **DFT Calculations:** Performing DFT calculations to determine topological parameters for all ligands would be computationally intensive and time-prohibitive, given the scope of our study. Additionally, integrating DFT-derived parameters with the AutoDock scoring function could introduce compatibility issues.
- **Force Fields:** Similarly, the use of external force fields would require additional parameter conversion steps and might not align perfectly with the AutoDock scoring function. The AutoDockTools parameterization is specifically tailored to the AutoDock scoring functions, ensuring consistency and reliability.

Utilizing AutoDockTools for ligand parameterization provided a streamlined and consistent approach that is directly aligned with the requirements and optimizations of the AutoDock suite. This methodology ensured reliable and reproducible docking results, forming a robust basis for our study’s conclusions. We hope this detailed explanation addresses the reviewer's concerns and clarifies our methodology. We are grateful for the opportunity to improve our manuscript based on the reviewer’s valuable feedback.

*Molecular Dynamics Simulations:*

*While the AutoDock results are appropriate and relatively solid, I recommend that the authors conduct additional molecular dynamics simulations. Using an effective platform such as* *GROMACS for these simulations could provide valuable insights into the stability of the formed complex. Molecular dynamics simulations would allow for observing how the docking complex behaves in a simulated environment and verifying its stability over time.*

**REPLY:** Thank you for your constructive comments.We sincerely appreciate the reviewer’s valuable suggestion regarding the incorporation of molecular dynamics (MD) simulations to further validate the stability of the docking complex. We acknowledge the significant insights that MD simulations can provide in terms of understanding the dynamic behavior and stability of protein-ligand interactions. However, due to the constraints related to the scope, timeline, and available resources for the current study, we are unable to perform additional MD simulations at this juncture. Our primary focus in this work has been to leverage robust docking methodologies, specifically through the AutoDock suite, to elucidate the initial binding interactions and affinities of the ligands. These results, we believe, provide a substantial contribution to our understanding of the ligand-receptor interactions within the framework of this study. While we acknowledge that MD simulations would offer additional layers of validation and dynamic insights, we have undertaken thorough validation using alternative approaches and methods that are well-established in the field. We are confident that the data and analyses presented in our manuscript provide a solid and valuable foundation for our conclusions. We fully recognize the importance of MD simulations and their potential to enrich our findings. As such, we intend to incorporate these simulations in future studies to extend and build upon the current research. This will enable us to explore the dynamic stability of the complexes in greater detail and validate the docking results further. We hope that the reviewer understands the constraints we are operating under and appreciates our position. We remain committed to advancing our research and integrating MD simulations in our subsequent investigations.
