# Supplementary material for: Cellular senescence-associated genes in rheumatoid arthritis: Identification and functional analysis
Source: PLoS One. 2025 Jan 16;20(1):e0317364. doi: 10.1371/journal.pone.0317364 (PMC11737674; doi:10.1371/journal.pone.0317364)
Supplement: Attachment — Submitted filename: review_PONE-D-24-02627_R2.docx [file pone.0317364.s009.docx]

**Dear Editor and Authors,**

First of all, I apologize for the delay in responding with my opinion on the review carried out by the authors. After reviewing the additions, based on the suggestions I previously made, some aspects of the article have been clarified, especially those related to the analysis conditions related to docking. Regarding my considerations on the possible improvements related to the use of an XGBoost classification architecture instead of RF, after thinking about it in detail, with the new explanations and comparisons with tests carried out with both architectures, I agree with the authors that the RF-based strategy seems to be the most appropriate in this case. Even so, I would like to point out that although minimizing the number of genes involved indeed translates into a potentially lower cost and "simplicity," taking into account the importance of these two factors, I think that it may be necessary to also mention the negative impact that this excessive minimization of genes involved can have on sensitivity and/or specificity, basically to fine-tune the elimination of possible false negatives. Thus, although perhaps a trade-off analysis could have been performed to see how sensitivity and specificity are affected as the number of selected genes is reduced, I understand that this task may be outside the clear objective presented in this incipient article, although perhaps it could be kept in mind for the future evolution of the work. In this sense, I think that perhaps reference could be made to this possible debate in the manuscript so that the need to examine in detail the compromise between the reduction of the number of genes involved and the possible effect on the sensitivity and specificity of the technique is made clear. Perhaps the use of XGBoost and the greater number of genes with detected involvement could be mentioned, although the three underlined ones are used in the end.

I appreciate that the information and details of the molecular structures in their bioinformatics version have been substantially increased, mentioning different databases and approximations that are used, such as the addition of hydrogens or the type of partial charges.

Finally, I appreciate that you have understood the good intentions and probabilities of significant findings using molecular dynamics results, and I fully understand the timeline and the specific purpose of the manuscript. I understand that it falls within the limits of this manuscript, but I consider it necessary to name docking as a possible evolution since there are very relevant issues, such as interaction through hydrogen bonds, that may be very poorly represented in docking and would require a detailed analysis of molecular dynamics to study in detail both the affinity and the binding and their mechanisms. In this sense, I consider it appropriate to mention it so that readers are aware of the need to answer some questions. All of this, of course, without implying a level of detail that conflicts with future work that the authors are planning or carrying out.

With these minor revisions that I propose, as far as my opinion is concerned, I consider that the article very pertinently complies with an originality and impact suitable to be presented to the scientific community. I thank you for taking my opinions into account and for giving me the opportunity to participate in the review of this valuable manuscript.
