# Supplementary material for: Cellular senescence-associated genes in rheumatoid arthritis: Identification and functional analysis
Source: PLoS One. 2025 Jan 16;20(1):e0317364. doi: 10.1371/journal.pone.0317364 (PMC11737674; doi:10.1371/journal.pone.0317364)
Supplement: Attachment — Submitted filename: Response to Reviewers .docx [file pone.0317364.s010.docx]

**Subject:** Re: Manuscript PONE-D-24-02627R2

Dear Dr. Selvaraj,

Thank you for providing the opportunity to revise our manuscript titled “Cellular Senescence-Associated Genes in Rheumatoid Arthritis: Identification and Functional Analysis.” We appreciate the reviewers’ and editor’s constructive comments, which have significantly improved our work. We have carefully addressed all points raised, and a detailed point-by-point response is attached. The revisions include:

- Ensuring data availability as per PLOS ONE guidelines.
- Updating the reference list for completeness and accuracy.

Please find the following documents submitted for your consideration:

- A rebuttal letter detailing our responses.
- A revised manuscript with changes tracked.
- A clean manuscript version without tracked changes.

Thank you again for considering our manuscript, and we look forward to your feedback on this revised submission.

**Response to Reviewer *#2*:**

Dear Reviewer,

Thank you for your detailed and thoughtful feedback on our manuscript. We appreciate your acknowledgment of the clarifications and improvements we have made. Below, we outline how we have addressed your suggestions:

*Trade-Off Between Gene Reduction and Model Performance:*

We have incorporated a discussion on the potential trade-offs between reducing the number of selected genes and the impact on sensitivity and specificity. While this analysis is outside the current scope, we agree it is an important consideration for future work. The revised manuscript as follows:

Page 16-17, section “**Discussion**”

“We acknowledge that minimizing the number of selected genes can potentially reduce costs and improve interpretability. However, this reduction might also negatively impact sensitivity and specificity, possibly increasing the risk of false negatives. Future studies could explore this trade-off in greater detail, performing comprehensive analyses to evaluate sensitivity and specificity as a function of gene selection thresholds. Furthermore, while we chose an RF-based strategy for its robustness in this context, exploring XGBoost or other advanced machine learning architectures in future studies could provide additional insights into the potential contribution of a larger gene set.”

*Mention of Molecular Dynamics:*

In response to your suggestion, we have added a brief discussion highlighting the limitations of docking and the potential for molecular dynamics to provide deeper insights into molecular interactions, particularly hydrogen bonding. The revised manuscript as follows:

Page 14, section “**Association between signature genes and drug sensitivity**”

“Molecular docking (AutoDock) provides a computationally efficient approach to evaluate molecular interactions; however, it may inadequately capture certain specific interactions, such as hydrogen bonding. Molecular dynamics simulations offer a more comprehensive framework for understanding binding affinities and mechanisms. In our future studies, we could leverage these simulations to complement and refine the insights gained from docking analyses.”

*Methodological Details:*

We have ensured that the additional details regarding databases, hydrogen addition, and charge types are clearly described in the methodology section. The revised manuscript as follows:

Page 9, section “**Association between signature genes and drug sensitivity**” in **Materials and Methods**

“Detailedly, the crystal structures of signature genes were downloaded from RCSB Protein Data Bank (PDB, https://www.rcsb.org/) [PMID: 24597646] and the 3D structure of small molecule compound was downloaded from the PubChem Compound database. The downloaded complex was embellished by PyMol2.3.0 to remove original water molecules and phosphates. Moreover, the AutoDock Tools 4.2.6 [PMID: 19399780] (https://autodock.scripps.edu/) was used to prepare receptors, including adding Gasteiger charges, merging non-polar hydrogen bonds and setting docking parameters. A box of 60*60*60-point grid (0.375-Å spacing between the grid points) and the affinity maps were generated and processed using AutoGrid 4.2 by default setting. For each docking case, 200 Lamarckian genetic algorithm runs were processed by default setting using AutoDock 4.2.6. To further confirm the binding of small molecule compound to the selected genes, we have also obtained the structures of proteins encoded by the selected genes from AlphaFold and then re-performed AutoDock. The top-scored hit was chosen and visualized for further analysis and the PyMOL was used to preparse all the molecular graphics.”

We sincerely appreciate your constructive comments, which have improved the quality of our manuscript. Thank you for your valuable input.

Sincerely,

You Ao

Department of Orthopaedics, The fifth hospital of Harbin, Harbin, Heilongjiang, P.R. China
